# Cues to Lying May be Deceptive: Speaker and Listener Behaviour in an Interactive Game of Deception

**DOI:** 10.5334/joc.46

**Published:** 2018-09-27

**Authors:** Jia E. Loy, Hannah Rohde, Martin Corley

**Affiliations:** 1 University of Edinburgh, Edinburgh, GB

**Keywords:** Deception, Communication, Pragmatics, Disfluency

## Abstract

Are the cues that speakers produce when lying the same cues that listeners attend to when attempting to detect deceit? We used a two-person interactive game to explore the production and perception of speech and nonverbal cues to lying. In each game turn, participants viewed pairs of images, with the location of some treasure indicated to the speaker but not to the listener. The speaker described the location of the treasure, with the objective of misleading the listener about its true location; the listener attempted to locate the treasure, based on their judgement of the speaker’s veracity. In line with previous comprehension research, listeners’ responses suggest that they attend primarily to behaviours associated with increased mental difficulty, perhaps because lying, under a cognitive hypothesis, is thought to cause an increased cognitive load. Moreover, a mouse-tracking analysis suggests that these judgements are made quickly, while the speakers’ utterances are still unfolding. However, there is a surprising mismatch between listeners and speakers: When producing false statements, speakers are less likely to produce the cues that listeners associate with lying. This production pattern is in keeping with an attempted control hypothesis, whereby liars may take into account listeners’ expectations and correspondingly manipulate their behaviour to avoid detection.

## Introduction

To tell a lie is to knowingly produce an utterance that is false. In producing such an utterance, a speaker’s behaviour may contain cues which signal the lack of truth. These cues might range from speech cues such as hesitations, speech disturbances, and changes in the pitch or rate of speech, to nonverbal cues such as blinking and hand gestures (DePaulo et al., 2003; Sporer & Schwandt, 2006). A listener may therefore try to take advantage of a number of cues when attempting to judge the veracity of a statement (Akehurst, Köhnken, Vrij, & Bull, 1996; Zuckerman, Koestner, & Driver, 1981).

The present study explores a range of potential behavioural cues to lying, in order to compare those produced by speakers with those attended to by listeners. Since face-to-face communication is inherently multimodal, we consider both verbal and nonverbal behaviour. We consider the following questions: In cases where speakers utter a literal untruth, do they produce perceptible evidence that they are lying; moreover, do listeners make use of these cues in order to infer the truth? This is of particular interest, since evidence from lie perception demonstrates that listeners hold strong beliefs regarding the discriminative value of many cue behaviours (Akehurst et al., 1996; see Zuckerman, Koestner, & Driver, 1981 for a meta-analysis), despite independent evidence from lie production to suggest that the actual cues that correlate with lying are weak (see Hartwig & Bond, 2011, for a meta-analysis).

We present a novel treasure-hunting game. In each turn, a speaker chooses whether to correctly name the location of some treasure or to lie, and a listener guesses where the treasure is hidden. In exploratory analyses of speakers’ utterances and gestures, we demonstrate which cues speakers tend to produce when lying, and which cues listeners interpret as evidence that a statement is false. In confirmatory analyses of time-locked mouse movements, we explore listeners’ sensitivities to the relevant cues in real time, allowing us to establish that their judgements emerge early, as the speakers’ utterances are still unfolding.

In the next section, we first discuss prior work examining speech cues to lying, looking at both the perception and production of cues, followed by a similar discussion on nonverbal cues. We compare models of deception that provide competing explanations regarding the presence and interpretation of behavioural cues.

### Behavioural cues to lying

A review of studies on the perception of speech cues to lying delineates a stereotypical image of a liar. Listeners expect liars to speak more slowly, pause longer, and speak with a higher pitched voice. These behaviours appear to carry perceptual relevance in both individual studies investigating lie perception (Vrij, 2000), as well as meta-analytic studies examining patterns across conditions (Hartwig & Bond, 2011; Zuckerman, DePaulo, & Rosenthal, 1981), and hold regardless of whether subjects are evaluating lie behaviour in themselves or in others (Zuckerman, Koestner, & Driver, 1981). Chief among the set of speech cues that listeners associate with lying are filled pauses, or verbalisations such as *um* and *uh* that mark hesitation on the speaker’s part. This interpretation is consistent with the belief that *um* and *uh* arise from production problems (Clark & Fox Tree, 2002; Fox Tree, 2007), a feature commonly associated with lying (Vrij, Edward, & Bull, 2001).

Production studies also frequently identify filled pauses as a behavioural correlate of lying. However, findings differ as to the direction of correlation. In line with listeners’ expectations, several studies report an increase in filled pause production when lying (Vrij et al., 2001; Vrij & Winkel, 1991). For example, Vrij and Winkel (1991) found that participants told to lie about a mock crime in a simulated police interview produced more filled pauses than those instructed to tell the truth. However, other studies using similar paradigms report a decrease in filled pauses (Vrij, 1995; cf. Arciuli, Mallard, & Villar, 2010), and yet others report no reliable difference between liars and truth-tellers (Granhag & Strömwall, 2002; Mann, Vrij, & Bull, 2002). To add to the uncertainty, some meta-analytic reviews of lie production studies note an increase in liars’ filled pauses (Zuckerman & Driver, 1985; Zuckerman, Koestner, & Driver, 1981), while others report no change in this behaviour (Hartwig & Bond, 2011).

Although some studies suggest that there is a diagnostic advantage for listeners who rely solely on verbal information (Vrij, 2008), others have shown that accuracy is higher when non-verbal cues are also taken into account (Vrij, Edward, Roberts, & Bull, 2000; Vrij & Mann, 2001). Experiments in which participants are asked to assess the veracity of speakers in police interview clips frequently find that cues such as gaze aversion and fidgeting are associated with falsehood (Mann, Vrij, & Bull, 2004; Vrij & Mann, 2001). These findings largely align with results from questionnaires investigating peoples’ beliefs about lying, which reveal that observers tend to interpret behaviours such as decreased eye contact and a higher frequency of adaptors (e.g., scratching, touching one’s hair or clothing, and other self-directed manipulations) as signs of dishonesty (Akehurst et al., 1996; Vrij & Semin, 1996; Zuckerman, Koestner, & Driver, 1981).

Turning to the actual nonverbal behaviours exhibited by liars, however, evidence from lie production research is less clear about the cues that correlate with lying. Take gaze aversion as an example: inconsistency can be observed even within the same subject. In an analysis of the true and false statements produced by a convicted murderer during two separate police interviews, Vrij and Mann (2001) found that the subject showed more gaze aversion whilst lying than while truth-telling in one interview, but less in another. Similarly, Granhag and Strömwall (2002) observed more adaptors, such as scratching or adjusting one’s clothing, in liars, while Vrij and Winkel (1991) observed fewer. Meta-analyses paint a similarly conflicted picture. Sporer and Schwandt (2007) report a decrease in hand movements and foot and leg movements in liars, while DePaulo et al. (2003) and Hartwig and Bond (2011) report no measurable difference in the two variables.

One possible reason for such disparate results, even across meta-analyses, is that the process of aggregating findings may conceal situational variations. In the case of gaze aversion, for example, Vrij and Mann (2001) tentatively attribute the inconsistency within their subject to the different styles of questioning employed by the officer in each interview, highlighting the potential influence of interlocutor attitude on a liar’s behaviour (cf. Anolli & Ciceri, 1997). In a similar vein, Vrij and Heaven (1999) demonstrate the impact of lie complexity on a liar’s speech behaviour: More complex lies such as having to fabricate a reason for stealing a satellite TV resulted in an increase in participants’ speech hesitation and speech disturbances, while cognitively simpler lies such as those about a person’s appearance resulted in a decrease. Although some meta-analytic studies have considered the effect of certain moderating factors such as lie content (Sporer & Schwandt, 2007) or a liar’s motivation to succeed (DePaulo et al., 2003; Hartwig & Bond, 2014), other factors, such as whether or not the participant was instructed to lie on cue (‘cued-lying’ paradigms) have been largely overlooked. This may be important to take into account, since natural lies are rarely cued and typically produced at free will. We return to the issue of how lies are elicited in experimental paradigms below.

### Models of speaker deception

There are two dominant hypotheses concerning cues that a speaker is lying. The first, the *cognitive hypothesis*, emphasises the cognitive complexity associated with the act of lying. This hypothesis proposes that lying requires more mental effort, which in turn impacts a liar’s behaviour (Sporer & Schwandt, 2006; Vrij, 2000). The second, the *attempted control hypothesis*, focusses on the stereotypes of deceit and corresponding impression management measures employed by liars (Vrij, 1995). Under this hypothesis, speakers are aware that their behaviour may reveal an intent to lie, and thus attempt to counteract potential exposure by controlling their speech and body language.

With regard to speech behaviour, the cognitive hypothesis could explain a higher frequency of speech disturbances in liars, due to the increased mental load of having to construct a convincing lie. Vrij and Heaven (1999) systematically manipulated the complexity of the lie that speakers had to tell, and showed that the frequency of speakers’ hesitations increased with lie complexity. This hypothesis receives further support from non-deception paradigms, which show that people engaged in cognitively complex tasks tend to speak more slowly and pause more (e.g., Goldman-Eisler, 1968; Kjellmer, 2003).

The attempted control approach, on the other hand, supports a decrease in liars’ speech disturbances. For example, Villar, Arciuli, and Mallard (2012) observed that the speech of a convicted murderer contained fewer *ums* during false utterances, for statements produced in both private and public domains. This hypothesis is reinforced by evidence that speakers are able to regulate several aspects of their behaviour when lying (DePaulo, Blank, Swaim, & Hairfield, 1992; Johnson, Henkell, Simon, & Zhu, 2008), and furthermore, that offering the right motivation (e.g., a monetary incentive) can reduce filled pause production to near zero levels (Boomer & Dittmann, 1964).

The two hypotheses can each account for some of the divergent results in liars’ nonverbal behaviour. Evidence from question-answer paradigms, for example, demonstrate that speakers avert their gaze due to the increased cognitive load of answering difficult questions, possibly in an attempt to reduce or avoid environmental stimulation (for example the face of the questioner; Doherty-Sneddon, Bruce, Bonner, Longbotham, & Doyle, 2002; Doherty-Sneddon & Phelps, 2005). Gesturing and hand movements have also been linked to cognitive load-reduction strategies that speakers employ as they think about what to say (Goldin-Meadow, Nusbaum, Kelly, & Wagner, 2001). The cognitive hypothesis could thus explain why liars may avert their gaze or move their hands more as a by-product of the mental load associated with constructing a lie. On the other hand, liars, aware of the cue potential of their actions, may try to control these very behaviours to avoid being caught. The attempted control hypothesis may explain why some researchers note that liars can come across as unusually rigid and inhibited as a result of over-controlling their behaviour (e.g., Vrij, 1995). This hypothesis is also often cited as a reason why studies consistently fail to identify reliable indicators of lying, as the discriminative potential of cues may diminish the more liars are able to effectively regulate their behaviour (Granhag and Strömwall, 2002; cf. Buller, Comstock, Aune, and Strzyzewski, 1989).

It should be noted that the two hypotheses are not necessarily mutually exclusive (cf. Vrij & Mann, 2004). For example, a liar may speak more slowly due to having to think hard, whilst appearing rigid as a result of trying to control their movements. The behavioural cues which arise would depend in part on the liar’s ability to manage various behaviours concurrently: Ekman and Friesen’s (1969) *leakage hierarchy hypothesis* proposes that some channels of communication are harder for speakers to control than others. They note, for instance, that people should be more successful at monitoring their facial behaviour (except for micro-expressions; cf. Ekman, 2001) when lying than monitoring their hands, feet or bodies, due to the social salience of facial expressions in communication (cf. Vrij et al., 2001). In a similar vein, speech cues to lying (with the exception of voice pitch) are frequently held to be more controllable than many aspects of nonverbal behaviour (Ekman, O’Sullivan, Friesen, & Scherer, 1991; Sporer & Schwandt, 2006). It is therefore possible that cognitive effort and attempted control could both concurrently influence different aspects of a liar’s behaviour.

### Eliciting lies

Past researchers have criticised the tendency for studies to rely on cued-lying paradigms, where speakers are directed to lie or tell the truth by means of a colour or some other form of cue (e.g., Burgoon & Floyd, 2000). While such cues have the advantage of permitting a balanced design, for example allowing for even numbers of true and false statements, such “instructed lies” may be problematic as they likely invoke different processes than do those produced under the speaker’s own volition. This issue was addressed in a recent neuroimaging study by Sip et al. (2010) which utilised a game paradigm where participants made truthful or false claims about a dice throw at will. Sip et al. observed that in contrast to previous cued-lying studies, false claims were not associated with activity in the dorsolateral prefrontal cortex (DLPFC). The authors attribute this to the fact that their task did not involve decision-making at the level of selecting appropriate responses in the current context, a process which typically invokes activity in the DLPFC (cf. Frith, 2000).

Relying on instructed lies may also undermine a speaker’s motivation to lie convincingly, raising doubts about the authenticity of the lie produced. DePaulo et al. (2003) compared studies in which speakers were offered inducements to succeed at lying to those which offered no special motivation, and found that cues were more pronounced in speakers that were motivated. In a similar vein, Bond and DePaulo (2006) observed across 20 studies that lies produced by motivated speakers were easier to classify than those produced by unmotivated speakers.

A related concern stems from the tendency for studies to elicit post-hoc judgements, where participants are asked to make a truth/lie discrimination judgement based on audio or video recordings of speakers (e.g., Hart, Fillmore, & Griffith, 2009; Loy, Rohde, & Corley, 2017). This approach removes listeners from the immediacy and interactivity that characterise a typical act of lying. For example, using an eye- and mouse-tracking paradigm, Loy et al. (2017) provide evidence that listeners’ judgments about whether a speaker was lying or telling the truth about a prize’s location were made on-line, almost as soon as they could determine which of the two possible locations the speaker was referring to. However, listeners in the study heard pre-recorded utterances in a comprehension-only task.

Evidence from joint action research highlights that a listener’s interpretation is closely linked to the act of interacting with another; for example, overhearers in the director-matcher task perform more poorly than matchers, who are actively engaged in conversation with the director (Schober & Clark, 1989). Listeners have also been found to follow instructions more accurately when produced in a dialogue rather than monologue setting (Fox Tree, 1999).

An over-dependence on post-hoc, uni-directional lie perception tasks thus raises the question of how generalisable results are to a real-life, interactive context. With this in mind, we designed the current study to address the limitations of cued-lying, non-interactive paradigms. We designed an experiment involving pairs of participants in a turn-based treasure-hunting game, in which speakers utter falsehoods at will, and listeners judge each utterance’s veracity in real-time. The main aim of the study was to explore the production and perception of speech and nonverbal cues to lying in a naturalistic, interactive paradigm that more closely approximates real-life deception. Our results replicate prior findings that show that listeners associate pauses with lying, in keeping with the cognitive hypothesis. However speakers themselves show no such link, instead revealing other behaviors during deception that are in keeping with the attempted control hypothesis. As a secondary aim, we recorded and analysed listeners’ mouse movements: The results confirm an earlier finding that judgements about a speaker’s veracity emerge during the earliest moments of comprehension (Loy et al., 2017), even under the demands of real-time interaction.

### Experiment

The experiment was designed as a two-person competitive game. Following Loy et al. (2017), each trial presented a pair of images, with participants told that treasure was hidden behind one of them. One player, a Speaker, also received an indication of which image concealed the treasure. Speakers described the location of the treasure to the other player, but were free to lie at will (by indicating the false location). The other player, a Guesser, used a mouse to click on one of the two images in an attempt to reveal the treasure. Guessers retained treasure that they correctly identified; Speakers retained treasure which Guessers failed to click on. As motivation to lie has been implicated as a moderator of liars’ behaviour (DePaulo et al., 2003; Sporer & Schwandt, 2007; although see Hartwig & Bond, 2014), we also attempted to vary Speakers’ motivation by presenting two levels of treasure—gold and silver coins, with different point values for each. However, no significant effects involving motivation were found in any analyses, and this manipulation is not discussed further.

We coded the speech and gestures produced by Speakers for nineteen potential cues to lying. We employed a process of exploratory modelling based on the Akaike Information Criterion (AIC; Akaike, 1973) to determine which behavioural measures were reliable in predicting Speakers’ veracity, and Guessers’ judgements of veracity. By comparing the speech and nonverbal cues that Speakers produced to those that influenced Guessers’ judgements, we made an explicit comparison of speakers’ behaviours and listeners’ expectations surrounding the cues to lying. By using the cues that influenced Guessers to analyse their mouse movements in real time, we were able to demonstrate that Guessers’ initial judgements of veracity are made as early as possible during an unfolding utterance.

## Method

### Participants

Twenty-four same-sex (5 male; 19 female) pairs of native British English speakers took part in the study. Due to the exploratory nature of the study, we were unable to estimate power and sample size ahead of time. The sample size of twenty-four dyads was determined in part due to the operational and logistical constraints of running such an experiment, and in part to match the sample size of Loy et al. (2017) (*n* = 22), which included a mouse-movement analysis comparable to that of the present design. Participants were all right-handed mouse users who reported no speech or hearing disorders. All provided informed consent in accordance with the University of Edinburgh Psychology Research Ethics Committee guidelines (reference no.: 214/1415-1). An additional 2 pairs of participants were tested but their data were excluded on the basis of speakers reporting post-test that they had deliberately manipulated their speech behaviour during the experiment.

Within each dyad, one member was assigned the role of Speaker (the potential liar) and the other the Guesser (the lie detector). All dyads were unacquainted prior to the study. Participants received £4 or course credit in exchange for participation. The winner of each dyad received an additional £1 cash reward.

### Materials and design

The stimuli consisted of 96 black-and-white line drawings of objects, presented in fixed pairs across 48 trials. Forty-eight original images were drawn from Snodgrass and Vanderwart’s (1980) data set. Slight modifications were then made to each image, forming image pairs consisting of two objects which were visually related (e.g., a camel with one/two humps). This was done with the aim of eliciting complex noun phrases from Speakers when naming an object, in order to provide longer utterances for analysis.

**Figure 1:**
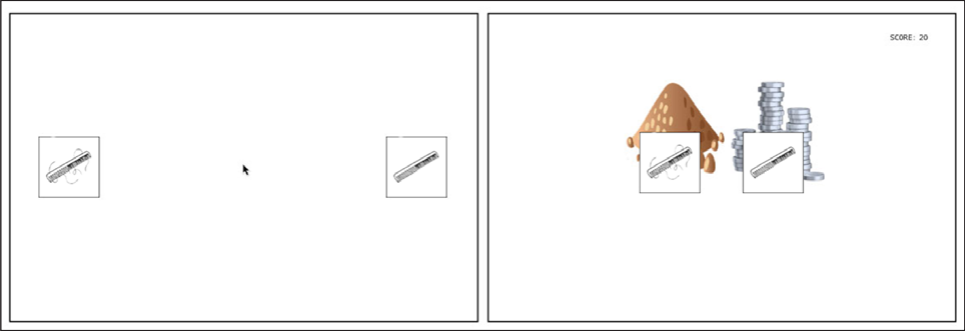
Example trial of the Guesser’s display (left) and Speaker’s display (right).

On each trial, one object within the pair was the target behind which the treasure was hidden, while the other served as a distractor. These were distinguished on the Speaker’s display by a pile of coins or a pile of dirt behind each object respectively (see Figure 1). The coins were either gold (worth 20 points) or silver (worth 5 points). Eight lists were created, counterbalancing the role of each object within each pair (target or distractor), position of the target (left or right), and the type of treasure associated with the target (gold or silver) across all 96 objects. The order of presentation of image pairs was randomised across dyads.

### Procedure

The roles of Speaker and Guesser were assigned at the start by drawing lots. Speakers were instructed to describe the location of the treasure on each trial to their partner, with the aim of misleading them into looking for the treasure in the wrong location. They were given no additional guidance, other than that they were free to indicate the false object if they wished. Their ultimate goal was therefore to mislead the Guesser—they could do this either through lying (identifying the false object with the expectation that the Guesser would believe them) or telling the truth (describing the correct object with the expectation that the Guesser would interpret it as a false claim). Guessers were instructed to click on the object that they believed concealed the treasure, with the knowledge that their partner might be lying to them. Both players were present at the same time for the instructions, and were thus aware of the role and motivation of the other.

**Figure 2:**
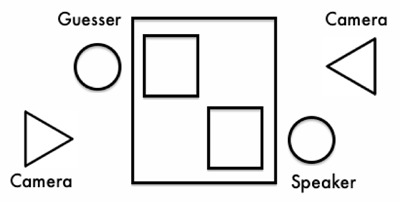
Diagrammatic setup of experiment.

The experiment was presented using OpenSesame Version 2.9.5 (Mathôt, Schreij, & Theeuwes, 2012) on 13 in. Apple Macintosh laptops. The Speaker and Guesser sat facing each other at diagonally opposite ends of a 24 × 36 in. table. This allowed positioning of video cameras at head level in front of each participant (see Figure 2).

Each trial began with a 1000 ms central fixation cross. This was then replaced by the image pair on each player’s screen. On Guessers’ displays, the objects were centred vertically and positioned horizontally left and right on the screen. The mouse cursor appeared at the centre at the same time as the images. On Speakers’ displays, the objects were set closer together to discourage Guessers from relying on Speakers’ gaze locations (see Figure 1).

Once a click had been detected on an object, both players saw a message indicating whether the Guesser had found the treasure. Guessers received points upon successfully locating the treasure; Speakers received points when the Guesser chose the wrong object. Players’ scores were cumulative over the course of the experiment. To incentivise dyads to compete against each other, a £1 cash reward was offered to the player with the higher score at the end.

A 3-trial practice session preceded the main experiment to familiarise dyads with the game, after which both players had the opportunity to ask questions before the game began. Trial progression was controlled from a separate computer by the experimenter, who initiated new trials when dyads were ready to continue after each one.

Participants’ facial and upper body gestures were filmed during the experiment. Speech was recorded separately using a Zoom H4N digital recorder. This provided us with audio and video recordings of the dialogue for each Speaker and Guesser pair from the beginning of each trial up until the point where Guessers clicked on an object. Guessers’ clicks on each trial (referent or distractor) were recorded, and provided an indication of whether they judged the Speaker to be lying or telling the truth. Guessers’ mouse pointer coordinates were also sampled at 50 Hz, with the aim of analysing the real-time trajectories of movements towards the images in response to Speaker cues (cf. Barr & Seyfeddinipur, 2010; Spivey & Dale, 2006).

## Results

### Data

Speech from each trial was transcribed and annotated in Praat (Boersma & Weenink, 2013). Speakers’ utterances on each trial were coded as truths or lies, with lies defined as an utterance that was factually incorrect, either about the treasure’s location (99.6% of all false utterances) or about the type of treasure—gold or silver coins (remaining 0.4%). Guessers’ responses were correspondingly coded as truth or lie judgements based on whether they clicked on the referent (object the Speaker named as concealing the treasure) or the distractor. Trials on which the Speaker was inconsistent in their commitment to the treasure’s location were excluded from analyses (0.3% of all utterances) since it was impossible to determine on these trials whether the Speaker intended to lie or tell the truth from the outset. These were cases in which the Speaker appeared to change their mind about where the treasure was hidden, such as in (1) where the images depicted a live and a dead flower:

**Table:** 

(1)	S: the treasure is not behind the flower that is not dying
	G: as in it’s behind the dead flower
	S: it’s behind the alive flower

The final dataset comprised 1,149 recorded utterances produced by 24 speakers. Video data from one participant was lost due to operator error; hence the final video dataset consisted of recordings of 1,101 trials from 23 speakers. Speakers were truthful 53.9% of the time (SE = 1.9, min = 35.4, max = 68.8) while Guessers judged 55.8% of the recorded utterances to be truthful (SE = 2.1, min = 33.3, max = 79.2). These figures are in line with the general trend in lie production and lie perception studies, which often note a global bias toward telling or expecting the truth (Vrij, 2000). The mean truth-lie discrimination accuracy for Guessers was 48.0% (SE = 1.4, min = 33.3, max = 60.4), with a 53.5% accuracy when Speakers were telling the truth and a 41.7% accuracy for Speakers’ deceptive utterances. This difference is again unsurprising given the overall tendency for Guessers to perceive utterances as truthful (cf. Bond & DePaulo, 2006).

### Annotation of utterances

The transcribed utterances were annotated for disfluencies by the first author and each disfluency was labelled for type. The following types of disfluency were identified: filled pauses, silent pauses, repetitions, restarts, substitutions, additions, and prolongations. To assess the reliability of the disfluency annotations, 20% of the speech data was randomly extracted and coded independently by a second coder. For all coding of speech as well as nonverbal data (described below), both coders were blind to the Speaker’s veracity and the Guesser’s response for each trial. Table 1 provides the kappa statistics for interrater agreement between the two coders.

**Table 1:** Descriptive statistics and Cohen’s Kappa (κ) between the two coders for the individual speech and gesture variables.

	raw count	mean (SD)	κ

**Speech variables** (*n* = 1, 149)			
Filled pauses	288	–	.95
Silent pauses	588	–	.97
Repetitions	55	–	.87
Restarts	109	–	.95
Substitutions	36	–	.95
Additions	12	–	1.0
Prolongations	334	–	.82
Utterance duration	–	3008.92 (1329.35)	–
Silent pause duration	–	651.65 (1080.5)	–
Speech syllable rate	–	3.82 (1.42)	–
**Gestures** (*n* = 1, 101)		–	
Head movements	651	–	.76
Hand movements	280	–	.92
Body movements	377	–	.87
Shoulder movements	26	–	.85
Lip/mouth movements	85	–	.50
Eyebrow movements	242	–	.83
Smiles/laughter	156	–	.81
Gaze	130	–	.95

Table 2 presents the correlations between Speakers’ truths, Guessers’ perception of utterances as truths, and the seven disfluency types, along with three continuous speech measures (described below). Since some types of disfluency accounted for very few observations in the dataset (see Table 1), the disfluencies were collapsed into four main categories. These were identified based on similar classification systems employed by existing studies (e.g., Hartsuiker & Notebaert, 2010; Merlo & Mansur, 2004; Shriberg, 1996). The categories are: (a) pauses, both filled (e.g., *uh, um* or *mm*) and silent; (b) repetitions; (c) repairs, where a verbalisation was interrupted and restarted or corrected with a substitution or addition; and (d) prolongations. Table 3 provides examples of the disfluencies in each category.

**Table 2:** Correlations between Speakers’ truths, Guessers’ perception of utterances as truths, and individual speech variables.

	1	2	3	4	5	6	7	8	9	10	11	12

1. Truths	1.00											
2. Perception of truths	0.08	1.00										
3. Filled pauses	0.12	–0.09	1.00									
4. Silent pauses	0.11	–0.17	–0.33	1.00								
5. Repetitions	0.01	0.01	–0.25	–0.42	1.00							
6. Restarts	0.19	–0.06	–0.30	–0.42	–0.20	1.00						
7. Substitutions	–0.07	–0.10	–0.07	–0.28	–0.21	0.03	1.00					
8. Additions	–0.12	0.11	–0.18	–0.24	0.03	–0.13	–0.19	1.00				
9. Prolongations	–0.01	–0.09	–0.17	–0.61	–0.19	0.00	0.18	–0.21	1.00			
10. Utterance duration	–0.05	0.07	0.31	0.56	0.18	0.28	0.07	0.08	0.47	1.00		
11. Silent pause duration	–0.03	0.09	0.17	0.59	0.23	0.17	0.05	0.07	0.35	–0.55	1.00	
12. Speech syllable rate	0.08	–0.06	–0.23	–0.68	–0.13	–0.13	–0.01	–0.03	–0.51	0.64	0.59	1.00

*Note.* Correlations are tetrachoric for associations between binomial variables (1–9); Pearson’s for associations between continuous variables (10–12); and point-biserial for associations between binomial and continuous variables. All correlations are conducted at the observation level and do not take participant or item dependencies into account.

**Table 3:** Disfluency categories and examples from data.

Disfluency category	Example

Pause	behind **um** the banana that’s not peeled
	behind the camel with **(0.32)** two humps
Repetition	behind the- **the** cut cake
Repair	**the money is th-** behind the one with the big tail fin
	behind the necklace which has beads coming- **falling** off it
	behind the open- **more** open book
Prolongation	behind **thee** leaf that looks like the ace on a pack of cards

The following continuous measures were also extracted from each utterance: duration of utterance; total silent pause duration within the utterance; and speech syllable rate (the number of perceptually salient syllables per second of speech). Speech onset latency was also measured, but is not reported here as Speakers did not always begin with task-related speech (e.g., commenting on the stimuli or making other irrelevant observations). A general combined measure of speech rate was computed by extracting the first factor of a principal components analysis (PCA) on the three measures of utterance duration, silent pause duration, and speech syllable rate. This component had an eigenvalue of 2.19 and explained 73% of the variance. The PCA was conducted in R (version 3.2.4; R Core Team, 2016) using the FactoMineR library (Lê, Josse, & Husson, 2008).

Video recordings of Speakers on each trial were annotated in Elan (Wittenburg, Brugman, Russel, Klassmann, & Sloetjes, 2006) for their nonverbal behaviour. The following gestures were identified: head movements, hand movements, body movements, shoulder movements, lip/mouth movements, eyebrow movements, smiling/laughter, and eye contact.^1^ This was operationalised as instances where the camera (positioned at head level next to the Guesser, see Figure 2) recorded Speakers looking up from their screen and at the Guesser. We did not code whether Guessers were directly fixating Speakers’ gestures because research on visual attention suggests that gestures are frequently perceived through peripheral vision, and listeners can often attend to something without fixating it (‘seeing without looking’; Gullberg & Kita, 2009). Videos for trials in which the Speaker was inconsistent regarding their commitment to the treasure’s location were excluded. Only gestures produced during the duration of the Speaker’s utterance were annotated. As with the speech data, 20% of the video data was extracted and coded independently by a second coder. Table 1 provides the kappa statistics for interrater agreement between the two coders.

Table 4 presents the correlations between Speakers’ truths, Guessers’ perception of utterances as truths, and the eight gesture variables. The gestures were categorised as one of three main categories, identified based on Ekman and Friesen’s (1969) system of classifying nonverbal behaviour. The categories are: (a) adaptors, encompassing self-oriented movements performed with little awareness and no message intent; (b) illustrators, defined as gestures designed to supplement or modify speech; and (c) affect displays, defined as gestures (primarily facial expressions) that function to convey specific emotions. A further category, eye contact (operationalised as described above), represented Speakers’ gaze behaviour. Table 5 presents specific examples of gestures in each category.

**Table 4:** Correlations between Speakers’ truths, Guessers’ perception of utterances as truths, and individual gestures.

	1	2	3	4	5	6	7	8	9	10

1. Truths	1.00									
2. Perception of truths	0.08	1.00								
3. Head movements	–0.07	–0.01	1.00							
4. Hand movements	–0.12	0.08	–0.22	1.00						
5. Body movements	–0.10	–0.09	–0.11	–0.08	1.00					
6. Shoulder movements	0.12	0.11	–0.16	–0.07	0.00	1.00				
7. Lip/mouth movements	0.11	–0.10	–0.22	0.16	–0.10	–0.25	1.00			
8. Eyebrow movements	0.01	0.03	–0.41	0.12	–0.08	–0.12	–0.33	1.00		
9. Smiles/laughter	0.07	0.16	–0.27	–0.17	–0.30	–0.02	–0.15	–0.17	1.00	
10. Eye contact	–0.07	0.01	–0.37	–0.57	–0.15	0.10	0.19	–0.11	–0.30	1.00

*Note.* All correlations are tetrachoric. Correlations are conducted at the observation level and do not take participant or item dependencies into account.

**Table 5:** Gesture categories and examples from data.

Gesture category	Example

Adaptor	Hand movements such as scratching one’s head, adjusting one’s clothing, clasping one’s hands etc.
	Body movements such as rocking forwards, backwards or sideways Postural adjustments such as slumping or straightening one’s back
Illustrator	Hand movements such as chopping motions to indicate a sliced carrot
	Head movements such as a head shake to indicate a tree with no fruit on it
Affect display	Eyebrow movements such as raised eyebrows to demonstrate surprise or furrowed brows to express concentration
	Mouth movements such as pursed lips to indicate thought Smiling or laughing during the utterance
Eye contact	Raising eyes from the screen to make eye contact with the Guesser

Each utterance was additionally coded for a disambiguation point: namely the earliest point during the utterance at which it could be determined which of the two images the Speaker was referring to as the treasure’s location. This provided a point to which Guessers’ mouse movements were time-locked for analysis. For the images in Figure 1, for example, the disambiguation point was determined as shown (in boldface) in each of the utterances in (2):

**Table:** 

(2)	behind the comb with **hairs** in it
	behind the **hairy** comb
	behind the comb with**out** any hair
	behind the comb that has **no** hair

Trials on which the disambiguation point could not be determined due to additional dialogue between Speakers and Guessers were excluded from this analysis (18.9% of all utterances). These were mainly cases where Guessers asked for clarification, such as in (3) or (4).

**Table:** 

(3)	S: the treasure is behind the candle that isn’t f- very melted
	G: isn’t very melted
	S: yeah the like fresh candle

**Table:** 

(4)	S: it’s behind the- the key that has the bit on the end
	G: as in the old-fashioned key
	S: they old-fashioned key, yeah

In cases such as these, although the Speaker’s initial utterance disambiguates between the two images, the additional dialogue makes it unclear as to when the Guesser actually established which image the Speaker intended to convey as the treasure’s location. To assess the reliability of the disambiguation point coding, 20% of the speech data was randomly extracted and coded independently by a second coder. Interrater agreement between the two coders was high, κ = .996, *p* = 0.

### Analysis

Statistical analyses were carried out in R using the lme4 package (Bates, Maechler, Bolker, & Walker, 2014). We were interested in how well each of the speech and gesture categories predicted whether (a) Speakers were telling the truth or lying, and (b) Guessers perceived Speakers to be telling the truth or lying. Logistic mixed-effects regression models were used to model the outcome variables of Speakers’ veracity (truth or lie) and Guessers’ response (truth or lie, based on whether they clicked on the referent or distractor) for each utterance.

Due to the exploratory nature of the analysis, and our goal of identifying variables of interest among a large set of predictors, we used the Akaike Information Criterion (AIC; Akaike, 1973) in a process of model evaluation to determine the best model given the data.^2^ The AIC value of a model is defined as –2*log*(ℒ) + 2*K*, where *log*(ℒ) is the maximised log-likelihood of the model and *K* is the number of estimable parameters (Burnham & Anderson, 2002). For small sample sizes, Hurvich and Tsai (1989) recommend a sample correction to the AIC:

{\rm AICc} = {\rm AIC} + \frac{{(2K(K + 1))}}{{(n - K - 1)}},

where *n* is the sample size and *K* and AIC are as defined above.

We used AICc to select the best-supported model from a set of competing models designed to explain the outcomes of Speaker veracity and Guesser response. We conducted separate analyses to examine the effects of speech and gesture categories on each dependent variable. We first determined for each dependent variable a set of candidate models. This comprised all possible additive combinations of the set of predictors for the given outcome. For the speech models these included the four disfluency categories and speech rate, yielding a total of 2^5^ = 32 potential models, including a null (intercept-only) model. For the gesture models these included the three gesture categories and eye contact, yielding a total of 2^4^ = 16 potential models, again including the null. All models included random intercepts for participants and items (defined as the target image concealing the treasure on that trial).^3^ All predictors other than speech rate were binary, and no distinction was made between one or more occurrences of the behaviour during the utterance.

For each candidate model within a set, we calculated (a) its AICc value; (b) its AICc difference with respect to the best model (ΔAICc); (c) its AICc weight (*w_i_*), which provides a measure of the conditional probability of the model (Akaike, 1978; Wagenmakers & Farrell, 2004); and (d) its evidence ratio (*ER_i_*), which represents the strength of evidence of favouring the best model over that model (Wagenmakers & Farrell, 2004). We also computed (e) the cumulative AICc weight (Σ*w_i_*) for individual parameters by summing the AICc weights across all models including that variable (Tables 6 and 7). This provides a strength of evidence measure for each parameter (cf. Arnold, 2010), and is scaled between 0 (weakest) and 1 (strongest). Formulae used to derive measures (c), (d), and (e) are given in Appendix A.

**Table 6:** Cumulative AICc weights (0 ≤ Σ*w_i_* ≤ 1) of speech model parameters for Speaker veracity and Guesser response.

Model parameter	Σ*w_i_*	

	Speaker veracity	Guesser response

pauses	0.61	0.79
repetitions	0.27	0.33
repairs	0.56	0.64
prolongations	0.43	0.36
speech rate	0.43	0.33

**Table 7:** Cumulative AICc weights (0 ≤ Σ*w_i_* ≤ 1) of gesture model parameters for Speaker veracity and Guesser response.

Model parameter	Σ*w_i_*	

	Speaker veracity	Guesser response

adaptors	0.81	0.27
affect displays	0.28	0.76
illustrators	0.36	0.30
gaze behaviour	0.32	0.27

When evaluating which speech or gesture categories were reliable in predicting an outcome, we considered the model with the lowest AICc, as well as candidate models with a ΔAICc of less than 2 with respect to that model (cf. Burnham & Anderson, 2002). Appendix B provides a complete list of all the models in each candidate set along with their associated AICc ranking, AICc weight and evidence ratio, as well as the final model output for the best-supported model (as determined by AICc_min_) from each candidate set.

### Speech cues

Table 6 presents the cumulative AICc weights of the speech parameters used to model Speaker veracity and Guesser response.

The best-supported model in estimating the effect of the speech variables on predicting Speakers’ veracity was the model containing only pauses. This model had an AICc weight of 0.108, indicating that it accounted for 10.8% of the total weight of all models in the candidate set, and ranked 0.70 AICc units above the second best-supported model. The model was 1.42 times more likely than the next best model to be the most parsimonious for the data, as indicated by the evidence ratio of the latter.

Model coefficients showed a positive relationship between pauses and veracity: Speakers were 1.3 times more likely to be telling the truth when their utterance contained a pause, *β* = 0.26, SE = 0.13, *p* = .04 (*e*^0.26^ = 1.30). Correlations between Speakers’ truths and individual speech cues show that both filled and silent pauses correlated positively with truth-telling (Table 2), suggesting that the relationship between pauses and veracity was driven by both forms of pauses.

Of the 32 candidate models, 8 were within 2ΔAICc of the best model and hence can be interpreted as competitive in predicting the given outcome. Of these 8 models, 6 incorporated pauses. Pauses also had the highest cumulative AICc weight of the 5 variables (Table 6), lending support to the influence this variable had in predicting outcome. The remaining 2 supported models incorporated only speech rate (ΔAICc = 1.36) and prolongations and speech rate (ΔAICc = 1.34) respectively.

For Guessers, the best-supported model in estimating the effect of the speech variables on response judgement was the model containing only pauses. This model accounted for 15.1% of the total weight of all models in the set, as indicated by its AICc weight, and ranked 0.26 AICc units above the second best-supported model. The model was 1.14 times more likely to be considered the best model for the given data than the second best model, based on the evidence ratio of the latter.

In contrast to Speakers, the model coefficients for Guessers showed a negative relationship between pauses and truth perception: Guessers were 0.67 times as likely to click on the referent (and therefore more likely to click on the distractor) when the utterance contained a pause, *β* = –0.39, SE = 0.13, *p* < .01. In other words, pauses were more likely to be associated with lying by Guessers. Correlations between Guessers’ truth perception and individual speech cues show that both filled and silent pauses correlate negatively with truth perception (Table 2), suggesting that the relationship between pauses and Guesser response was driven by both types of pauses.

Of the candidate models, 6 were within 2ΔAICc of the best model. All 6 competitive models incorporated pauses. The influence of pauses on Guessers’ responses is also supported by the cumulative AICc weights, which show that pauses had the highest weight of all 5 speech variables (Table 6).

Together, these results highlight the role of pauses in predicting Speaker veracity and Guesser response. Analysis of the best-supported model for each candidate set, the subset of models deemed competitive, and the cumulative AICc weights of the individual model parameters provide unified evidence in support of this variable. Model coefficients also indicate a difference in the direction of effect on each outcome variable: Although pauses were an index of truth-telling in Speakers, they were associated with lie judgements in Guessers.

### Gesture cues

Table 7 presents the cumulative AICc weights of the gesture parameters used to model Speaker veracity and Guesser response.

The best-supported model in estimating the effect of gestures on predicting Speakers’ veracity was the model containing only adaptors. This model accounted for 26.3% of the total weight of all models in the set, as indicated by its AICc weight, and ranked 1.27 AICc units above the second best-supported model. The model was 1.88 times more likely to be the best model for the given data than the next best model, as indicated by the evidence ratio of the latter.

Model coefficients showed that Speakers’ veracity varied with their production of adaptors: Speakers were 0.75 times as likely to be telling the truth (and therefore more likely to be lying) when their utterance was accompanied by an adaptor, *β* = –0.29, SE = 0.13, *p* = .02. Of the three gesture cues that constituted adaptors, hand and body movements correlated negatively with Speakers’ truths while shoulder movements correlated positively (Table 4), suggesting that the relationship between adaptors and veracity was driven primarily by Speakers’ hand and body movements.

Three of the 16 candidate models were within 2ΔAICc of the best model, all of which incorporated adaptors. Adaptors also had the highest cumulative AICc weight of all 4 gesture variables on Speaker veracity (Table 7).

For Guessers, the best-supported model for the effect of gestures on response was the model containing only affect displays. This model accounted for 28.1% of the total weight of all models in the set, as indicated by its AICc weight, and ranked 1.64 AICc units above the second best-supported model. The model was 2.27 times more likely to be the best model for the data than the next best model, as indicated by the evidence ratio of the latter.

Model coefficients showed a positive relationship between affect displays and truth perception: Guessers were 1.34 times more likely to click on the referent when the utterance was accompanied by an affect display, *β* = 0.29, SE = 0.14, *p* = .04. In other words, Speakers’ lack of affect displays were more likely to be associated with lying by Guessers. Of the gestures that comprised affect displays, lip movements correlated negatively with truth perception while eyebrow movements and smiles/laughter correlated positively, suggesting that the relationship between affect displays and Guesser response was driven primarily by the latter two behaviours.

Of the 16 candidate models, 3 were within 2ΔAICc of the top model, all of which incorporated affect displays. Affect displays also had the highest cumulative AICc weight of all 4 gesture variables on Guessers’ responses (Table 7).

Results from the gesture analyses thus highlight different variables in predicting Speaker veracity and Guesser response. Evidence from the best-supported model, the subset of competitive models, and the cumulative AICc weights from each analysis show that adaptors had the greatest influence in explaining veracity, while affect displays had the greatest influence in explaining response. Model coefficients reveal a difference in the direction of effect of each variable on its outcome: Adaptors were an index of falsehood in Speakers, while affect displays were interpreted as a sign of truth-telling by Guessers.

### Mouse movements

A remaining question concerns the timecourse of Guessers’ judgements. Loy et al. (2017) demonstrated that, within 400 ms of the onset of a target name, mouse movements diverged, such that listeners were slower to move the pointer towards the referent if its name followed a disfluent pause, suggesting that in these cases, listeners were attracted to the distractor. However, that experiment made use of scripted and pre-recorded disfluent utterances. It is of interest whether listeners are similarly quick to make such cue-based judgements in the more natural dialogue situation afforded by the present experiment.

In the current study, an analysis based on the objects that listeners eventually selected shows that their responses are primarily influenced by (a) spoken pauses; and (b) gestures signalling affect. In two further analyses (one each for speech and gesture cues, as above), we compared mouse movements in response to utterances including these behaviours to those in response to all other relevant utterances.

For each utterance in each analysis, we determined the timepoint at which it first became clear to which of the two displayed images the Speaker intended to indicate as the location of the concealed treasure. Relative to this disambiguation point, we looked at the degree to which the mouse pointer was moving towards, or away from, the referent over time. We coded mouse movements in 20 ms bins, recording for each time bin the total number of pixels travelled along the *x*-axis, towards either the referent or the distractor. For each bin we then calculated a proportion-of-movement measure, defined as the *x* distance the mouse pointer moved toward the given object divided by the total distance travelled up until that time bin (regardless of *x* direction).

We analysed mouse movements over a time window beginning from the disambiguation point of each utterance to 800 ms post-disambiguation (cf. Loy et al., 2017). Since we were interested in the effect of cue behaviours on Guessers’ early inferences of veracity, we excluded from this analysis trials on which the relevant behaviour occurred post-disambiguation (28 out of 525 utterances (5.3%) containing a pause; 39 out of 273 utterances (14.3%) accompanied by an affect display). Models were fit using empirical logit regression (Barr, 2008). The dependent variable was the difference between the e-logit of the proportion of movements toward the referent and toward the distractor, and fixed effects were time and the behavioural cue of interest (pauses or affect gestures). All models included by-subjects and by-items random intercepts and slopes for both predictors.

**Figure 3:**
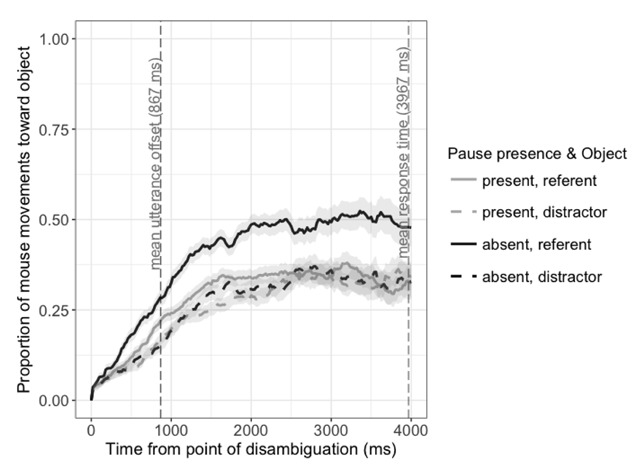
Proportion of cumulative distance travelled towards each object in response to utterances including a pause, compared to other utterances, from 0 to 4000 ms after the disambiguation point. Proportions are based on the total cumulative distance covered by the mouse pointer over time. Shaded areas represent ±1 standard error of the mean.

Figure 3 shows the degree of mouse movements (in terms of distance travelled) towards each object until 4000 ms after the disambiguation point, for utterances including pauses compared to all other utterances. Listeners’ mouse movements showed a smaller bias toward the referent following utterances that contained a spoken pause, as indicated by a time by pause interaction, *β* = –0.56, SE = 0.18, *t* = –3.19.

**Figure 4:**
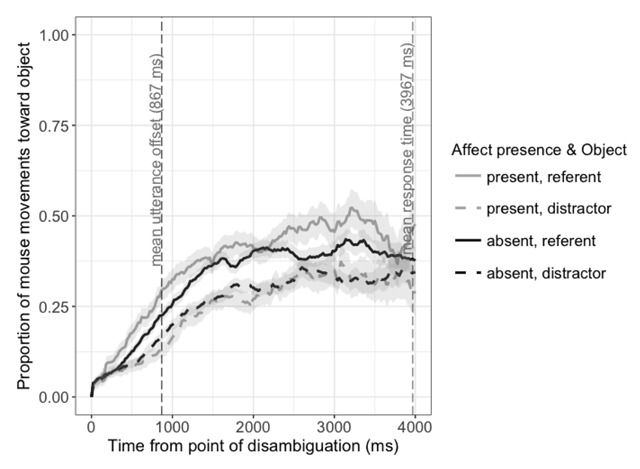
Proportion of cumulative distance travelled towards each object in response to utterances accompanied by affect gestures, compared to other utterances, from 0 to 4000 ms after the disambiguation point. Proportions are based on the total cumulative distance covered by the mouse pointer over time. Shaded areas represent ±1 standard error of the mean.

Figure 4 shows the amount a listener moves the mouse pointer towards each object until 4000 ms after the disambiguation point, for utterances accompanied by affect gestures compared to all other utterances. Listeners’ mouse movements showed a larger bias toward the referent following utterances accompanied by an affect display, as indicated by a time by affect interaction, *β* = 1.46, SE = 0.20, *t* = 7.33.^4^

Taken together, these analyses show that judgements are made early: pause disfluencies and a lack of affect displays very quickly bias listeners toward inferring that an utterance is false. In other words, the manner in which an utterance is delivered affects not only how it is eventually interpreted, but how listeners interpret it in real time. We return to this point in the Discussion below.

## Discussion

In the present study, we investigated the production and perception of speech and nonverbal cues to lying. We were interested in which cues speakers produced while deliberately saying something that was factually correct or incorrect, and whether those same cues were used by listeners to judge the veracity of an utterance. To that aim, we designed a task that elicited both true and false utterances from Speakers, as well as responses from listeners (Guessers) which indexed whether they believed that each utterance was in fact truthful. This allowed us to obtain judgements on utterances as participants interacted in real time, unlike previous studies which have employed post-hoc methods of judgement (e.g., Hart et al., 2009). Importantly, we were able to observe Guessers’ judgements as they unfolded, by measuring the position of the mouse pointer over time as they listened to the Speakers.

Our experiment demonstrated three things. First, and unsurprisingly, we observed a general bias towards truthfulness in both Speakers and Guessers. This aligns with the existing literature on deception, which highlights a tendency for speakers to tell the truth (Benus, Enos, Hirschberg, & Shriberg, 2006; Vrij et al., 2001), and a tendency for listeners to interpret utterances as truthful (Levine, Park, & McCornack, 1999). Second, and surprisingly, we found a mismatch between the cues that Speakers produced when lying, and the cues that Guessers used to infer falsehood, for both verbal cues and gestures. Third, by tracking Guessers’ mouse movements, we were able to show that judgements of veracity happen very fast: Where relevant verbal or gestural cues are produced, mouse movements are influenced by the image that is *not* being described almost as soon as the image that *is* being described can be uniquely identified. To that end, our results provide a conceptual replication of Loy et al.’s (2017) findings in an ecological context involving real-time interaction between speakers and listeners.

We return to the core finding of a mismatch between Speakers and Guessers below. First, we briefly discuss a methodological contribution of the current paper: That of using an AICc-based approach to exploratory analysis.

### Exploratory methods

The need to include a large number of predictors in a set of analyses is often the case with research on disfluencies (e.g., Merlo & Mansur, 2004; Shriberg, 1996) or deception (e.g., Granhag & Strömwall, 2002; Vrij, Akehurst, & Knight, 2006), as well as in areas such as discourse processing (e.g., Quek et al., 2000; White, 1989). For naturalistic studies like these, the AICc-based approach used here represents an alternative to traditional methods of analysis, which have often involved independent testing of individual predictors, leading to problems associated with multiple inference (cf. Curran-Everett, 2000).

AICc-based model selection results in sets of models which can be seen as evidence of which variables among a set are informative in explaining an outcome and which are not (cf. Snipes & Taylor, 2014). It is important to note that this approach does not provide definitive evidence for one model over others; rather, the AICc trends in each analysis should be taken as combined evidence to support conclusions drawn from the data.

As with any methodology, however, AICc has its drawbacks. In particular, the 2ΔAICc rule used to establish a subset of competitive models can be criticised as being an arbitrary cutoff, akin to the *p* < .05 significance rule in traditional null hypothesis testing; and indeed, various interpretations of ΔAIC (or ΔAICc) values have been adopted by different researchers (see Murtaugh, 2014, p. 615, for a summary). In partial mitigation of this problem, Burnham, Anderson, and Huyvaert (2011) recommend taking into account model likelihoods, evidence ratios, and consideration of all candidate models in the set to make an informed overall inference. With that in mind, we computed AICc weights and evidence ratios for all models, as well as cumulative AICc weights for individual model parameters. From our results, it is clear that these quantitative measures provide converging evidence for the importance of the variables that emerged as significant in the best-supported models.

### Mismatches between Speakers and Guessers

Our exploratory modelling revealed mismatches between the cues that reliably predicted Speakers’ veracity and those that Guessers attended to in judging whether an utterance was true. In terms of spoken cues, the best model for Speakers revealed pauses to be a significant predictor: Speakers were more likely to be telling the truth when their utterance contained a pause, either filled or silent. Although pauses also emerged as a significant predictor in the best Guesser model, this relationship was in the opposite direction: Guessers interpreted Speakers’ utterances containing pauses as untruthful.

A disconnect between Speakers and Guessers was again observed in the pattern observed for gestures. Speakers were more likely to produce adaptors in the context of a lie. Guessers, however, were not sensitive to adaptor production: Instead, they tended to infer falsehood from the absence of affect displays from the Speaker.

The results for Guessers corroborate findings from existing lie perception research (Akehurst et al., 1996; Zuckerman, Koestner, & Driver, 1981), and paint a portrait of a liar as one embroiled in a state of difficulty, either due to the cognitive burden of having to formulate a lie (Vrij et al., 2000), or that of experiencing various negative emotions associated with the act of lying (Ekman, 2001). Similarly, research into gesture highlights facial cues such as decreased smiling or an unfriendly facial expression as indicators of lying (Vrij et al., 2006; Zuckerman, DePaulo, & Rosenthal, 1981). Together, then, our findings suggest that Guessers’ impressions about lying reflect the cognitive hypothesis, where producing untrue utterances requires additional mental effort, resulting in cue behaviours indicative of this load (Sporer & Schwandt, 2006; Vrij, 2000).

Speakers, however, appear not to exhibit the behaviours which Guessers associate with falsehood. Since Speakers are also often in the position of listening to others (and, presumably, of making inferences concerning their honesty) it may be that their behaviour is evidence for the attempted control hypothesis: Speakers attempt to suppress the cues that they know Guessers will use to infer dishonesty (Vrij, 1995). The fact that they are successful in doing this is consistent with research that shows that under the right circumstances, speech disfluency can be reduced or eliminated (Boomer & Dittmann, 1964; Broen & Siegel, 1972).

Although Speakers managed to reduce their disfluency production while lying, our gestural analysis suggests that they were less successful at controlling their nonverbal behaviour: specifically they produced more adaptors when lying. This disparity is in line with Ekman and Friesen’s (1969) leakage hierarchy hypothesis, which alludes to certain channels of communication being harder to control than others. Our Speakers’ behaviours thus suggest a trade-off where the attempt to control their speech cues led to a decreased ability to control their nonverbal behaviour, resulting in cue leakage via their body language.

The behaviours we observed in Speakers and Guessers thus support both the cognitive hypothesis and attempted control hypothesis. However, the fact that Guessers appear to rely on the former while Speakers are influenced by the latter suggests a disconnect between expectations and reality surrounding cues to lying. This parallels an observation in the literature that listeners hold consistent beliefs about cues to deception, despite separate evidence from lie production to suggest that the actual cues liars exhibit are relatively weak (Hartwig & Bond, 2011). The fact that we observed a similar result in the context of a naturalistic paradigm where liars and lie-perceivers interacted in real-time suggests that these (misguided) beliefs that listeners hold may be so ingrained that they are difficult to overcome, even in the face of contradictory evidence.

Most of the available literature on deception compares situations where a speaker tells the literal truth to those in which their utterances are factually incorrect (e.g., Hart et al., 2009; Vrij et al., 2001). An aspect of the present paradigm, however, is that Speakers could have attempted to mislead Guessers by telling the truth, in the hope of being disbelieved (a ‘double bluff’). It is possible that reasoning that a truthful statement will be disbelieved places a greater mental load on Speakers than does straightforward lying (cf. Sutter, 2009). This may then lead to an increase in certain cue behaviours such as speech disturbances and adaptors. It is less clear, however, why double-bluffing would be predicted to lead to differential effects in speech and in gesture. Given that the ultimate aim of the Guesser is to divine the literal truth or otherwise of the Speaker’s statements, and that the Speaker must ultimately produce statements which are literally either true or false, we have elected to restrict our analyses to first-order veracity (although we note that double-bluffing remains an interesting avenue for further research). From this simpler perspective, it seems unsurprising that Speakers in our experiment showed a decrease in pause behaviour but an increase in adaptors when lying—an asymmetry resulting from an inability to control all aspects of their behaviour equally (cf. DePaulo et al., 2003).

Another aspect of the present paradigm is mutual awareness, whereby Guessers knew that Speakers might lie, while Speakers knew that Guessers were assessing whether they had lied. This was a necessary design feature, since in order to determine which cues Guessers relied on, they first had to be told to expect a potentially dishonest speaker. Although we did not manipulate the level of awareness in either player, a few studies have indicated that a liar’s behaviour may be moderated by how suspicious a listener appears (Anolli & Ciceri, 1997), while a listener may perceive cues differently depending on how suspicious they are of the speaker (Granhag & Strömwall, 2000). Future research could move on to investigate the role of mutual knowledge and suspicion in the context of an interactive paradigm such as ours.

### Mouse movements

Even if double-bluffing could be used to account for Speaker behaviour, it does not appear that Guessers are employing very sophisticated reasoning in deciding on the veracity of each utterance. This is because, as demonstrated in the analyses of mouse movements, Guessers are affected by the cues that lead them to infer truth or falsehood at the earliest possible moment: As soon as it becomes clear which object the Speaker is referring to, the mouse pointer moves towards it less quickly if the utterance contains a spoken or gestural cue associated with lying.

The finding that a spoken pause influences real-time interpretation is important for two reasons. First, it is consistent with earlier research using pre-recorded utterances (King, Loy, & Corley, 2018; Loy et al., 2017). By using naturally-occurring dialogue, we are able to rule out the possibility that participants in the earlier studies were sensitised to patterns in a restricted set of recorded items. Second, these findings establish a direct link between the signals associated with lying (pauses, adaptors) and the online interpretation of Speakers’ utterances. In other words, Guessers’ interpretations of Speakers’ utterances are updated in real time, based on the manner in which they are delivered. This may explain in part why Guessers don’t appear to second-guess Speakers. Since Guessers are also often in the position of speaking, it would be reasonable to assume that they were sensitive to Speakers’ attempts to control the cues they produced (in other words, Guessers could conclude via a process of inference that the *absence* of cues associated with cognitive effort signals lying). The fact, however, that Guessers’ responses seem to be determined at the earliest possible moment suggests that these auditory and visual cues may be difficult to override. This, admittedly speculative, interpretation would suggest that listeners’ judgements have ‘primacy’ (such that pauses are routinely associated with lying), and speakers’ behaviour is predicated on these ingrained associations.

## Conclusions

Extending previous studies which have examined cues to lying from a unidirectional perspective, we were able to investigate the simultaneous production and perception of cues using an interactive framework. This enabled us to simulate authentic, real-world deception in a controlled, yet relatively natural context. By allowing Speakers to choose when to lie and when to tell the truth, we also avoided problems associated with cued-lying paradigms, where the directed nature of the task may undermine the authenticity of the lies (cf. Spence, Kaylor-Hughes, Farrow, & Wilkinson, 2008). This provides ecological validity to Speakers’ utterances, their behavioural cues accompanying those utterances, and Guessers’ responses to those cues. The results we observed suggest that a liar’s behaviour is influenced not only by the act of conceiving a lie, but by the expectations that listeners may have regarding the speaker’s speech and gestures. Thus, the study highlights the importance of considering the interactive dimension in lie production and lie perception paradigms in order to contribute to a more complete understanding of the psychological dynamics that shape an act of deception.

## Additional Files

The additional files for this article can be found as follows:

10.5334/joc.46.s1 Appendix A. AICc and Evidence Ratio formulae.

10.5334/joc.46.s2 Appendix B. Details of Models.

## Data Availability

To avoid disclosing personal information about participants, original audio and video recordings from the experiment are not publicly available. However full post-transcription data, mouse-tracking data, and scripts for analysis can be found at osf.io/auj5b/.

## Footnotes

Gaze aversion is typically analysed as a behavioural correlate of lying. However, due to the setup of our study which encouraged participants to look at their screen more than their interlocutor, gaze aversion was the norm, hence annotation took into account instances where eye contact was made.

AIC is a tool for comparing multiple models on a given outcome, allowing for comparison of non-nested models which cannot be handled by traditional hypothesis testing. AIC model ranking provides a useful method for evaluating the relative importance of multiple potential predictors (cf. Arnold, 2010), as is often the case in exploratory studies (e.g., Pagano & Arnold, 2009; Snipes & Taylor, 2014). AIC estimates information loss based on Kullback-Leibler (K-L; 1951) information quantity, which can be seen as the discrepancy between the model and full reality (Burnham et al., 2011; Wagenmakers & Farrell, 2004). AIC model selection aims to minimise K-L information loss: Hence, smaller values, which denote less information loss, are preferred. Accordingly, the ‘best’ model among a set of competing models can be determined by selecting the model with the lowest AIC value (AIC*_min_*). It should be noted that raw AIC values on their own are meaningless; it is the differences in scores between models, the Δs, that provide a point for comparison. The difference in AIC for a given model *i* with respect to the best model is calculated by ΔAIC = AIC*_i_* – AIC*_min_*.

The inclusion of random slopes led to non-convergence for many of the models with 3 or more predictors. Slopes were therefore omitted from all models to maintain a consistent random effects structure.

Our mouse movement analysis was based on the subset of trials on which cue behaviours were initiated prior to the disambiguation point. We note, however, that the same analysis taking all trials into account show the same pattern of results, although effect sizes are reduced, *β* = –0.47, SE = 0.17, *t* = –2.74 for pauses; *β* = 1.07, SE = 0.19, *t* = 5.77 for affect displays. This supports our conclusion that Guessers’ early inferences about utterance veracity were influenced by the verbal and nonverbal cues Speakers produced during their delivery.
