# Supplementary material for: Cues to Lying May be Deceptive: Speaker and Listener Behaviour in an Interactive Game of Deception
Source: J Cogn. 2018 Sep 27;1(1):42. doi: 10.5334/joc.46 (PMC6634475; doi:10.5334/joc.46)
Supplement: Appendix A. — AICc and Evidence Ratio formulae. [file joc-1-1-46-s1.pdf]

Appendix A

AICc and Evidence Ratio formulae

Formulae used to derive AICc weights and evidence ratios of models, and cumulative AICc weights of model parameters:

AICc weight: $w_i = \frac{exp(-\frac{1}{2}\Delta\text{AICc}_i)}{\sum_{j=1}^{J} exp(-\frac{1}{2}\Delta\text{AICc}_j)}$

Evidence ratio: $ER_i = \frac{w_{best}}{w_i}$

Cumulative AICc weight of parameter p: $\sum w_i = \sum_{i=1}^{P} w_i$,

where i denotes the current model, J is number of models in the candidate set, w_{best} is the AICc weight of the best model, and P is the number of candidate models in the subset of models containing parameter p.
