# Supplementary material for: Cues to Lying May be Deceptive: Speaker and Listener Behaviour in an Interactive Game of Deception
Source: J Cogn. 2018 Sep 27;1(1):42. doi: 10.5334/joc.46 (PMC6634475; doi:10.5334/joc.46)
Supplement: Appendix B. — Details of Models. [file joc-1-1-46-s2.pdf]

Appendix B

Details of Models

Summary of AICc results for models exploring the effect of speech variables on predicting Speaker veracity. Models are ranked according to differences in Akaike's Information Criterion corrected for small sample sizes (AICc).

Model[a]	AICc	ΔAICc	w_i[b]	ER_i[c]
y ∼ ps + ε	1583.04	0.00	0.108	1.00
y ∼ ps + pl + ε	1583.73	0.70	0.076	1.42
y ∼ ps + rp + ε	1583.91	0.88	0.070	1.55
y ∼ pl + sr + ε	1584.38	1.34	0.055	1.95
y ∼ sr + ε	1584.39	1.36	0.055	1.97
y ∼ ps + rp + pl + ε	1584.74	1.71	0.046	2.35
y ∼ ps + pl + sr + ε	1584.75	1.71	0.046	2.35
y ∼ ps + sr + ε	1584.82	1.78	0.044	2.44
y ∼ ps + rr + ε	1585.02	1.99	0.040	2.70
y ∼ 1 + ε	1585.04	2.00	0.040	2.72
y ∼ rp + ε	1585.05	2.01	0.040	2.73
y ∼ rp + sr + ε	1585.30	2.26	0.035	3.09
y ∼ rp + pl + sr + ε	1585.64	2.60	0.030	3.69
y ∼ ps + rr + pl + ε	1585.73	2.70	0.028	3.85
y ∼ ps + rp + sr + ε	1585.84	2.80	0.027	4.06
y ∼ ps + rr + rp + ε	1585.87	2.83	0.026	4.12
y ∼ ps + rp + pl + sr + ε	1586.12	3.07	0.023	4.64
y ∼ rr + pl + sr + ε	1586.32	3.28	0.021	5.16
y ∼ rr + sr + ε	1586.36	3.32	0.021	5.26
y ∼ ps + rr + pl + sr + ε	1586.68	3.64	0.018	6.18
y ∼ ps + rr + rp + pl + ε	1586.72	3.68	0.017	6.31
y ∼ ps + rr + sr + ε	1586.78	3.74	0.017	6.49
y ∼ pl + ε	1586.82	3.79	0.016	6.64
y ∼ rp + pl + ε	1586.83	3.80	0.016	6.67
y ∼ rr + ε	1587.04	4.01	0.015	7.41
y ∼ rr + rp + ε	1587.06	4.03	0.014	7.49
y ∼ rr + rp + sr + ε	1587.23	4.20	0.013	8.15
y ∼ rr + rp + pl + sr + ε	1587.55	4.52	0.011	9.57
y ∼ ps + rr + rp + sr + ε	1587.77	4.74	0.010	10.67
y ∼ ps + rr + rp + pl + sr + ε	1588.02	4.98	0.009	12.08
y ∼ rr + pl + ε	1588.82	5.79	0.006	18.06
y ∼ rr + rp + pl + ε	1588.85	5.82	0.006	18.32

[a] y = Speaker veracity, ps = pauses, rr = repetitions, rp = repairs, pl = prolongations, sr = speech rate, ε = error term (random intercepts for subjects and items)
[b] AICc weight (relative likelihood of model based on AICc value)
[c] Evidence ratio (likelihood of favouring the best model over the current model i)

Model output for best-supported model for the effect of speech variables on Speaker veracity.

Model of pauses on Speaker veracity				
**Fixed effects**				
	Estimate	SE	z value	p value
(Intercept)	0.16	0.08	1.96	.05
pauses	0.26	0.13	2.01	.04
**Random effects**				
		Variance	SD	
participants	(Intercept)	0.05	0.23	
items	(Intercept)	0.10	0.31	
No. of obs: 1149, groups: participant, 24; item, 96				

Summary of AICc results for models exploring the effect of speech variables on predicting Guesser response. Models are ranked according to differences in Akaike's Information Criterion corrected for small sample sizes (AICc).

Model[a]	AICc	ΔAICc	w_i[b]	ER_i[c]
y ∼ ps + ε	1569.21	0.00	0.151	1.00
y ∼ ps + rp + ε	1569.47	0.26	0.132	1.14
y ∼ ps + sr + ε	1570.80	1.60	0.068	2.22
y ∼ ps + pl + ε	1570.88	1.67	0.065	2.31
y ∼ ps + rr + ε	1570.92	1.71	0.064	2.35
y ∼ ps + rp + pl + ε	1571.07	1.86	0.060	2.54
y ∼ ps + rr + rp + ε	1571.07	1.86	0.059	2.54
y ∼ rr + rp + pl + ε	1571.33	2.12	0.052	2.89
y ∼ ps + rr + sr + ε	1572.38	3.17	0.031	4.88
y ∼ sr + ε	1572.44	3.24	0.030	5.05
y ∼ ps + rr + pl + ε	1572.57	3.37	0.028	5.38
y ∼ ps + rr + pl + sr + ε	1572.64	3.43	0.027	5.57
y ∼ ps + pl + sr + ε	1572.69	3.48	0.026	5.71
y ∼ rp + sr + ε	1572.72	3.51	0.026	5.79
y ∼ ps + rp + pl + sr + ε	1572.84	3.63	0.025	6.15
y ∼ ps + rp + sr + ε	1573.07	3.86	0.022	6.90
y ∼ rr + sr + ε	1574.04	4.83	0.013	11.20
y ∼ rp + ε	1574.07	4.86	0.013	11.36
y ∼ rp + pl + ε	1574.18	4.97	0.013	12.03
y ∼ rp + pl + sr + ε	1574.24	5.03	0.012	12.39
y ∼ ps + rr + rp + sr + ε	1574.28	5.08	0.012	12.67
y ∼ pl + sr + ε	1574.30	5.10	0.012	12.80
y ∼ ps + rr + rp + pl + ε	1574.41	5.20	0.011	13.47
y ∼ ps + rr + rp + pl + sr + ε	1574.60	5.40	0.011	14.85
y ∼ pl + ε	1575.72	6.51	0.006	25.94
y ∼ 1 + ε	1575.73	6.53	0.006	26.17
y ∼ rr + rp + sr + ε	1575.92	6.71	0.005	28.68
y ∼ rr + rp + pl + sr + ε	1575.95	6.75	0.005	29.19
y ∼ rr + rp + ε	1575.97	6.76	0.005	29.38
y ∼ rr + pl + sr + ε	1576.01	6.81	0.005	30.04
y ∼ rr + pl + ε	1577.67	8.46	0.002	68.82
y ∼ rr + ε	1577.72	8.52	0.002	70.71

[a] y = Guesser response, ps = pauses, rr = repetitions, rp = repairs, pl = prolongations, sr = speech rate, ε = error term (random intercepts for subjects and items)
[b] AICc weight (relative likelihood of model based on AICc value)
[c] Evidence ratio (likelihood of favouring the best model over the current model i)

Model output for best-supported model for the effect of speech variables on Guesser response.

Model of pauses on Guesser response				
**Fixed effects**				
	Estimate	SE	z value	p value
(Intercept)	0.24	0.78	3.06	$< .01$
pauses	-0.39	0.13	-2.95	$< .01$
**Random effects**				
		Variance	SD	
participants	(Intercept)	0.06	0.24	
items	(Intercept)	0.00	0.00	
No. of obs: 1147, groups: participant, 24; item, 96				

Summary of AICc results for models exploring the effect of gesture variables on predicting Speaker veracity. Models are ranked according to differences in Akaike's Information Criterion corrected for small sample sizes (AICc).

Model[a]	AICc	ΔAICc	w_i[b]	ER_i[c]
y ∼ ad + ε	1516.03	0.00	0.264	1.00
y ∼ ad + il + ε	1517.30	1.27	0.140	1.88
y ∼ ad + ey + ε	1517.69	1.66	0.115	2.29
y ∼ ad + af + ε	1517.94	1.91	0.102	2.60
y ∼ ad + il + ey + ε	1518.68	2.64	0.070	3.75
y ∼ 1 + ε	1519.14	3.11	0.056	4.73
y ∼ ad + af + il + ε	1519.28	3.25	0.052	5.08
y ∼ ad + af + ey + ε	1519.53	3.50	0.046	5.75
y ∼ il + ε	1520.30	4.27	0.031	8.47
y ∼ ey + ε	1520.45	4.42	0.029	9.13
y ∼ ad + af + il + ey + ε	1520.61	4.58	0.027	9.87
y ∼ af + ε	1521.15	5.12	0.020	12.91
y ∼ il + ey + ε	1521.23	5.20	0.020	13.49
y ∼ af + il + ε	1522.32	6.29	0.011	23.19
y ∼ af + ey + ε	1522.42	6.39	0.011	24.44
y ∼ af + il + ey + ε	1523.25	7.22	0.007	36.93

[a] y = Speaker veracity, ad = adaptors, af = affect displays, il = illustrators, ey = eye contact, ε = error term (random intercepts for subjects and items)
[b] AICc weight (relative likelihood of model based on AICc value)
[c] Evidence ratio (likelihood of favouring the best model over the current model i)

Model output for best-supported model for the effect of gesture variables on Speaker veracity.

Model of adaptors on Speaker veracity				
**Fixed effects**				
	Estimate	SE	z value	p value
(Intercept)	0.18	0.08	2.11	.04
adaptors	−0.29	0.13	−2.26	.02
**Random effects**				
		Variance	SD	
participants	(Intercept)	0.08	0.28	
items	(Intercept)	0.06	0.23	
No. of obs: 1101, groups: participant, 23; item, 96				

Summary of AICc results for models exploring the effect of gesture variables on predicting Guesser response. Models are ranked according to differences in Akaike's Information Criterion corrected for small sample sizes (AICc).

Model[a]	AICc	ΔAICc	w_i[b]	ER_i[c]
y ∼ af + ε	1508.12	0.00	0.281	1.00
y ∼ af + il + ε	1509.76	1.62	0.124	2.27
y ∼ ad + af + ε	1510.08	1.97	0.105	2.67
y ∼ af + ey + ε	1510.08	1.97	0.105	2.67
y ∼ 1 + ε	1510.36	2.24	0.092	3.06
y ∼ ad + af + il + ε	1511.72	3.60	0.046	6.06
y ∼ af + il + ey + ε	1511.76	3.65	0.045	6.20
y ∼ ad + af + ey + ε	1512.06	3.95	0.039	7.19
y ∼ il + ε	1512.24	4.12	0.036	7.87
y ∼ ey + ε	1512.37	4.25	0.033	8.39
y ∼ ad + ε	1512.37	4.25	0.033	8.39
y ∼ ad + af + il + ey + ε	1513.73	5.62	0.017	16.60
y ∼ il + ey + ε	1514.26	6.14	0.013	21.55
y ∼ ad + il + ε	1514.26	6.14	0.013	21.57
y ∼ ad + ey + ε	1514.39	6.27	0.012	23.01
y ∼ ad + il + ey + ε	1516.28	8.16	0.005	59.21

[a] y = Guesser response, ad = adaptors, af = affect displays, il = illustrators, ey = eye contact, ε = error term (random intercepts for subjects and items)
[b] AICc weight (relative likelihood of model based on AICc value)
[c] Evidence ratio (likelihood of favouring the best model over the current model i)

Model output for best-supported model for the effect of gesture variables on Guesser response.

Model of affect displays on Guesser response				
**Fixed effects**				
	Estimate	SE	z value	p value
(Intercept)	0.23	0.90	2.57	.01
affect displays	0.29	0.14	2.05	.04
**Random effects**				
		Variance	SD	
participants	(Intercept)	< 0.01	< 0.01	
items	(Intercept)	< 0.01	< 0.01	
No. of obs: 1101, groups: participant, 23; item, 96				
